# Survival of stroke patients after introduction of the ‘Dutch Transmural Protocol TIA/CVA’

**DOI:** 10.1186/1471-2296-14-74

**Published:** 2013-06-04

**Authors:** Leonie de Weerd, Feikje Groenhof, Boudewijn J Kollen, Klaas van der Meer

**Affiliations:** 1 Department of General Practice, University of Groningen, University Medical Center Groningen, Antonius Deusinglaan 1, 9713 AV Groningen, The Netherlands

**Keywords:** Stroke, Survival, General practice, Healthcare, Prevention

## Abstract

**Background:**

Earlier research showed that healthcare in stroke could be better organized, aiming for improved survival and less comorbidity. Therefore, in 2004 the Dutch College of General Practitioners (NHG) and the Dutch Association of Neurology (NVN) introduced the ‘Dutch Transmural Protocol TIA/CVA’ (the LTA) to improve survival, minimize the risk of stroke recurrence, and increase quality of life after stroke. This study examines whether survival improved after implementation of the new protocol, and whether there was an increase in contacts with the general practitioner (GP)/nurse practitioner, registration of comorbidity and prescription of medication.

**Methods:**

From the primary care database of the Registration Network Groningen (RNG) two cohorts were composed: one cohort compiled before and one after introduction of the LTA. Cohort 1 (n = 131, first stroke 2001–2002) was compared with cohort 2 (n = 132, first stroke 2005–2006) with regard to survival and the secondary outcomes.

**Results:**

Comparison of the two cohorts showed no significant improvement in survival. In cohort 2, the number of contacts with the GP was significantly lower and with the nurse practitioner significantly higher, compared with cohort 1. All risk factors for stroke were more prevalent in cohort 2, but were only significant for hypercholesterolemia. In both cohorts more medication was prescribed after stroke, whereas ACE inhibitors were prescribed more frequently only in cohort 2.

**Conclusion:**

No major changes in survival and secondary outcomes were apparent after introduction of the LTA. Although, there was a small improvement in secondary prevention, this study shows that optimal treatment after introduction of the LTA has not yet been achieved.

## Background

The prevalence of stroke in general practice in the Netherlands is 13.3 in 1000 men and 13.4 in 1000 women [1]. Additionally, within 5 years after stroke, 50% of patients will die and 30-50% will experience another stroke [2].

A stroke has considerable impact on the patient’s life and his/her family [2,3]. Stroke can cause various neurological deficits, including paralysis, loss of vision, aphasia, spatial disorder, disturbance in thinking, difficulty in swallowing and incontinence [4]. Moreover, stroke has cognitive, behavioral, emotional and social consequences [5] and, in the chronic phase, negatively affects quality of life [3,4].

Studies have shown that healthcare in stroke patients could be better organized, that more thrombolysis treatment should be given and that more admissions to a stroke unit would result in better survival and less comorbidity [6-9]. It became increasingly obvious that secondary prevention (e.g. controlling blood pressure, cholesterol and glucose) is also very important. Treatment of cardiovascular/cerebrovascular risk factors reduces the risk of stroke, in particular hypertensive treatment. Moreover, consistent measurements to control whether treatment is effective are essential in stroke prevention programs [10]. All these findings emphasized that specific treatment is required for stroke patients.

A transmural stroke service, in which neurologists and general practitioners (GPs) participate, would allow proper care to be delivered in the appropriate setting. Patients should be quickly referred to the hospital and the GP is responsible for secondary prevention at home [11]. To improve survival after stroke and minimize the risk of stroke recurrence, the Dutch College of General Practitioners (NHG) and the Dutch Association of Neurology (NVN) introduced the ‘Dutch Transmural Protocol TIA/CVA’ (the LTA) in 2004 [12]. It also offers the GP and neurologist the advice to provide quality healthcare at the right time, without compromising the continuity of care.

The LTA protocol states that when acute neurological symptoms are apparent, the GP should visit the patient immediately so that thrombolytic treatment can be given within 3 hours after the symptoms started. Other indications for an emergency visit are unconsciousness, worsening of neurological symptoms, or when the event is so worrying for the patient or his/her environment that delay is not justified. Visiting the patient at home can be postponed if the situation is stable and no thrombolysis is possible [12].

The protocol recommends that agreements should be made about healthcare between the local hospital and GPs. In general, when a GP suspects an ischemic stroke, treatment with a platelet inhibitor or a coumarin derivative is started the same day and patients are admitted to a hospital (preferably a stroke unit). The protocol states that the neurologist is responsible for the remaining necessary medication, and for starting rehabilitation and secondary prevention in hospital. After discharge from hospital, the GP continues this treatment.

Antihypertensive medication is prescribed to patients with hypertension 2 weeks after the patient has stabilized. The protocol advises the prescription of statins to patients with a total cholesterol of 3.5 mmol/L or higher [12]. For treatment of high glucose levels, the NHG protocol ‘Diabetes mellitus type 2’ must be followed [13]. Health education should be performed by a neurologist, but the GP should also discuss risk factors, lifestyle changes and use of medication with the patient. This is important because patients regard the GP and neurologist as their main source of information [12,14,15].

GPs are responsible for assisting in the coordination of rehabilitation and post-hospital care of patients at home. The neurologist has to provide a discharge letter with treatment advice and a risk profile within 1 week after discharge from hospital to ensure that quality aftercare is possible [12,15].

The purpose of this study is to compare the survival of patients one year after stroke before and after the introduction of the LTA. We were not interested in establishing a causal relationship in survival before and after stroke due to the introduction of LTA, but only to show changes in survival occurring between pre- and post LTA time periods. We expected survival to be better in stroke patients after the introduction of the LTA, because follow-up and secondary prevention is implemented more regularly when complying more strictly with the LTA. Secondary outcomes include survival at 2-year follow-up, difference in the number of contacts with the GP/nurse practitioner, and difference in secondary prevention (e.g. prescription of medication, comorbidity, risk factors). We expected the number of contacts with the GP and the prescription of medication and comorbidities to be higher, because the LTA recommends that GPs check and monitor patients more regularly after stroke.

## Methods

### Study design and setting

In this registry study, the database of the Registration Network Groningen (RNG) was used. This primary care-based network was established in 1989, and consists of three group practices in the northern part of the Netherlands with about 20 GPs and about 30,000 patients [16]. The RNG is a validated register [17]. All contacts, diagnoses, referrals and prescriptions are registered in the RNG using the International Classification of Primary Care (ICPC) [18]. Anonymous patient data were used according to the privacy assignments by the RNG. The study was in agreement with the regulations for publication of patient data and, therefore, no further approval was required from the Medical Ethical Committees of the University Medical Centre Groningen.

### Participants and data collection

The study includes two cohorts of patients, one cohort compiled before and one after introduction of the LTA. Inclusion criteria were patients who had a first stroke in 2000–2001 (cohort 1) and in 2005–2006 (cohort 2). These patients had to be registered in the general practice for at least one year and were followed for two years after stroke. No additional selection criteria were applied. A total of 263 patients were included. Details on history, risk factors, mortality, morbidity, medication and referrals were obtained from the RNG.

### Statistical analysis

For statistical analysis SPSS 15 for Windows (SPSS Inc., Chicago) was used. Statistical significance was set at p < 0.05 (two-sided). To test differences between groups the Student’s *t*-test was used for normal distributed (continuous) variables and the Mann–Whitney *U* test was used for not normal distributed continuous, ordinal scaled or count variables. The Chi-square test was used for independent observations of nominal or dichotomous variables. The Kaplan-Meier method was used to estimate the survival distributions and the log-rank test was used to compare differences in survival between the groups [19,20].

## Results

### Baseline characteristics

A total of 263 patients were included: 131 patients in cohort 1 (first stroke 2000–2001) and 132 patients in cohort 2 (first stroke 2005–2006). Table 1 provides details on baseline characteristics: there were no significant differences between the two groups.

**Table 1:** Baseline characteristics of the study population

**Variables**	**Cohort 1 (%)**	**Cohort 2 (%)**	**p-value**
Patients included	131	132	
Gender			
*- Men*	72 (55)	59 (45)	0.096*
*- Women*	59 (45)	73 (55)	
Age, in years: average [range]	69.82 [19–105]	70.86 [31–103]	0.565†
Risk factors present before stroke/History			
*- K85 (high blood pressure without hypertension)*	8 (6)	9 (7)	0.815*
*- K86/87 (hypertension)*	34 (26)	31 (23)	0.643*
*- T93 (hypercholesterolemia)*	2 (2)	3 (2)	0.658*
*- T90 (diabetes)*	17 (13)	16 (12)	0.834*
*- K91 (arteriosclerosis)*	2 (2)	1 (1)	0.557*
*- K89 (TIA)*	5 (4)	9 (7)	0.278*
Average number of contacts (consults and visits) with general practice in the year preceding stroke			
*- Contact moments GP*	5.40	5.76	0.914‡
*- Consult GP*	3.48	5.76	0.346‡
*- Visits GP*	2.46	3.17	0.811‡
*- Contacts nurse practitioner*	1.00	1.01	0.319‡
Average exposure time^§^ in days	528	554	0.256‡

*Pearson’s Chi-square test, †Independent *T*-test, ‡ Mann–Whitney test. ^§^Exposure time: time during which patients were registered in a general practice during the study.

### Survival

**Figure 1:**
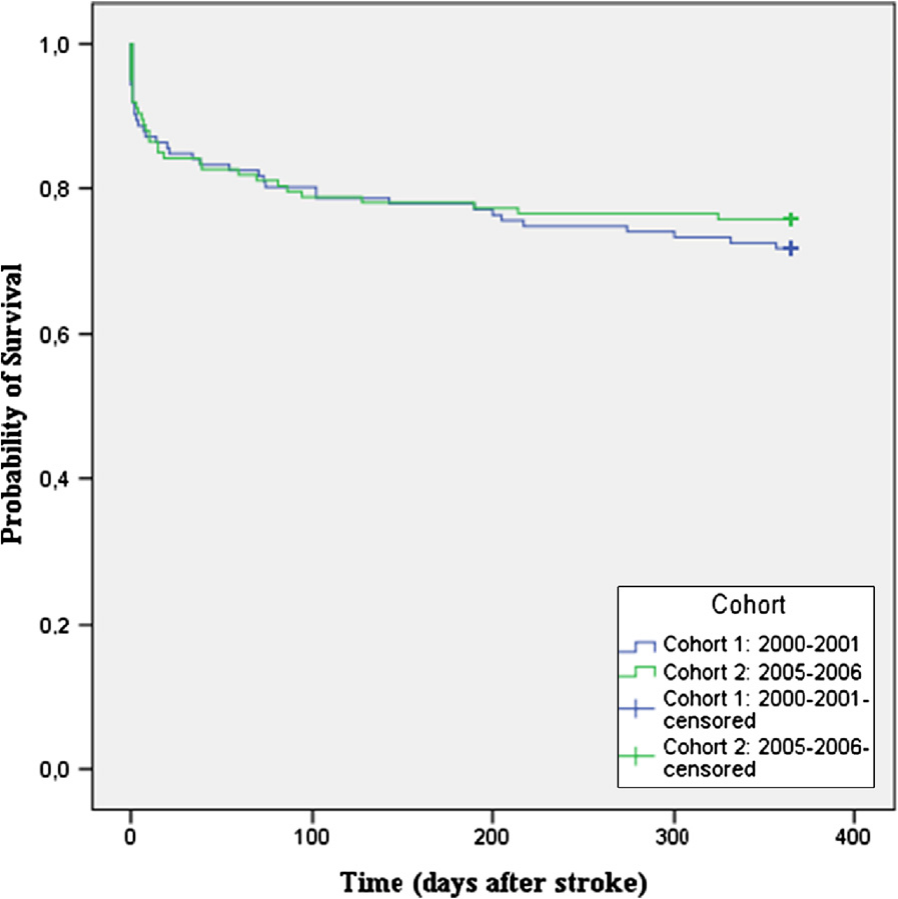
Survival at one-year follow-up.

**Figure 2:**
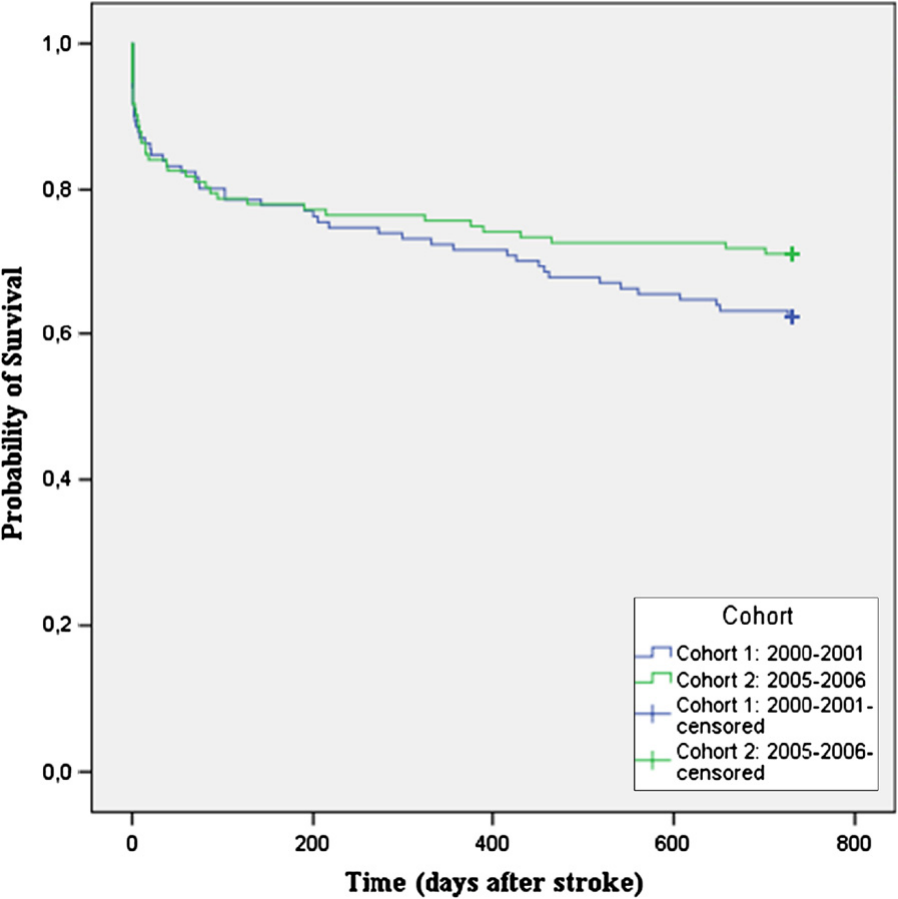
Survival at two-year follow-up.

Both cohorts were followed for two years, during which time some patients died. There was no significant difference in survival between the two cohorts at one-year follow-up (p = 0.511) (Figure 1) or at two-year follow-up (Figure 2) (p = 0.188).

Table 2 shows the percentage of patients that died in both cohorts. In cohort 1 more men and women died than in cohort 2; however, the difference is not significant. In both cohorts more patients died with increasing age.

**Table 2:** Number (%) of deceased patients during the two-year follow-up

**Deceased**	**Cohort 1 (%)**	**Cohort 2 (%)**	**p-value**^**#**^
Men			
- related to stroke*	8 (11)	6 (10)	0.862
*- within one year*	17 (24)	14 (24)	0.987
*- within two years*	24 (33)	18 (31)	0.730
Women			
- related to stroke*	6 (10)	6 (8)	0.698
*- within one year*	20 (34)	18 (25)	0.244
*- within two years*	25 (42)	20 (27)	0.071
0-60 years			
- related to stroke*	4 (3)	2 (2)	0.523
*- within one year*	4 (3)	2 (2)	0.523
*- within two years*	5 (4)	3 (2)	0.619
0-80 years			
- related to stroke*	10 (8)	6 (5)	0.306
*- within one year*	21 (16)	15 (11)	0.280
*- within two years*	27 (21)	17 (13)	0.091
Deceased related to stroke	14 (11)	12 (9)	0.665
Deceased during one-year follow-up	37 (28)	32 (24)	0.461
Deceased during two-year follow-up	49 (37)	38 (29)	0.138

*Related to first stroke: death within 3 days. ^#^Pearson’s Chi-square test.

### Healthcare in general practice

For healthcare consumption of patients in general practice, a distinction was made between the consults and visits with the GP and/or nurse practitioner. There were no significant differences between healthcare consumption of the two cohorts with the exception of the average number of contacts with the nurse practitioner (Table 3).

**Table 3:** Healthcare provided by general practice during two-year follow-up

**Variables**	**Cohort 1 (range)**	**Cohort 2 (range)**	**p-value**^**#**^
Median number of contacts with GP (consults/visits) per patient per year	8.0 (0–44)	7.0 (0–36)	0.801
Median number of consults per patient per year	2.0 (0–31)	2.0 (0–30)	0.746
Median number of home visits per patient per year	2.0 (0–33)	1.0 (0–30)	0.230
Median number of contacts with nurse practitioner per patients per year	0.0	0.0 (0–17)	**<0.001**

^#^ Mann–Whitney test.

### Secondary prevention

Theoretically, the LTA should lead to an increase in the number of ICPC codes in cohort 2 compared to cohort 1 (Table 4). Table 4 shows that all ICPC codes are more frequently applied in cohort 2 than in cohort 1; however, the difference is significant only for hypercholesterolemia.

**Table 4:** Number (%) of patients with different risk factors included in the prescription register and/or journal two years after stroke

**ICPC**	**Cohort 1 (%)**	**Cohort 2 (%)**	p-value^*^
K85 (high blood pressure without hypertension)	3 (2.3)	8 (6.1)	0.127
K86/87 (hypertension)	54 (41.2)	63 (47.7)	0.288
T93 (hypercholesterolemia)	24 (18.3)	45 (34.1)	**0.004**
T90 (diabetes)	26 (19.8)	31 (23.5)	0.474
K91 (arteriosclerosis)	1 (0.8)	2 (1.5)	0.566

^*^Pearson’s Chi-square test.

### Medication

With the introduction of the new protocol more attention is paid to secondary prevention and the prescription of medication, e.g. aspirin and statins. Table 5 presents data on the number of patients who were prescribed several types of medication in the year preceding the stroke and in the following two years post stroke.

**Table 5:** **Number of patients who were prescribed several types of medicine from one year before stroke (*Before *) until two years post stroke (*After*)**

**Medication**	**Number of patients in Cohort 1 (%)**			**Number of patients in Cohort 2 (%)**		
	***Before***	***After***	p-value*	***Before***	***After***	p-value*
Antithrombotics	50 (38.2)	85 (64.9)	**<0.001**	56 (42.4)	103 (78.0)	**<0.001**
Diuretics	55 (33.6)	36 (27.5)	0.165	47 (35.6)	49 (37.1)	0.798
Beta-receptor blocker	35 (26.7)	46 (35.1)	0.120	49 (37.1)	48 (36.4)	0.898
Calcium channel blocker	14 (10.7)	21 (16.0)	0.116	18 (13.6)	13 (9.8)	0.339
Medication influencing renin-angiotensin system	20 (15.3)	30 (22.9)	0.243	40 (30.3)	58 (43.9)	**0.022**
Antidiabetics	20 (15.3)	19 (14.5)	0.964	24 (18.2)	28 (21.2)	0.536
Cholesterol-lowering medication	15 (11.5)	23 (17.6)	**0.035**	39 (29.5)	68 (51.5)	**<0.001**

^*^ Pearson’s Chi-square test.

The results show that in both cohorts significantly more cholesterol-lowering medication and antithrombotics were prescribed after stroke. Also, only in cohort 2 significantly more medication affecting the renin-angiotensin system was prescribed after stroke.

## Discussion

This study examined whether the survival of stroke patients changed after the introduction of the ‘Dutch Transmural Protocol TIA/CVA’ , and whether the number of contacts with the GP/nurse practitioner, registration of comorbidity, and prescription rate of medication increased.

The two cohorts were well matched regarding the number of patients, age, gender and comorbidity.

After introduction of the LTA there was no significant difference in survival between cohort 1 and 2. We expected survival to improve after introduction of the LTA because the GPs could use the FAST test, i.e. a checklist in which patients are sent to hospital directly after telephone contact without causing any delay [12,21]. An explanation for this result might simply be that the LTA does not lead to improved survival, despite rapid and effective patient care. Another possibility is that the situation envisaged with the LTA was not achieved, perhaps due to problems associated with the implementation of thrombolysis and stroke unit treatment [22,23].

Although survival is slightly better in cohort 2 (Figures 1 and 2), our results show that there was no significant improvement in long-term survival after introduction of the LTA; a longer follow-up period might perhaps reveal a significant difference as both curves tend to diverge progressively over time.

The number of patient contacts in general practice was expected to increase after introduction of the LTA, because the LTA recommends GPs to aim for intensive rehabilitation and to monitor secondary prevention in stroke patients [12]. This should lead to an increase in the number of GP contacts, especially in the first year after stroke. There was a slight increase in contacts with the nurse practitioner, a nurse practitioner assists the GP in care for chronic patients [24], including stroke patients. This might be explained by the recent introduction of nurse practitioners for cardiovascular risk management in GP offices. Although there was an increase in contacts with the nurse practitioner this was not the case for the number of GP contacts and visits. A possible explanation is the establishment of GP centers, which probably means that visits by the GP in the evenings/weekends are no longer necessary [25]. Moreover, there is a tendency towards more telephone contacts and fewer home visits [26]. Another explanation is that insufficient attention is paid to rehabilitation by GPs, implying the stricter implementation of the LTA may still be beneficial. Exposure time (defined as number of days during which patients were registered in a general practice) was the same in both groups.

According to the LTA, secondary prevention in stroke patients is important, especially for the GP [12]. This should lead to an increase in the number of risk factors in cohort 2 compared to cohort 1. The data indeed showed an increase, but only the increase of hypercholesterolemia is significant; this might be because (in 2003) treatment of stroke patients (without elevated cholesterol) with statins proved to be effective [12,27]. However, whether secondary prevention has in fact improved remains debatable. A future questionnaire study among GPs and patients might provide more insight into secondary prevention after implementation of the LTA.

Implementation of the LTA was expected to increase use of medication to treat risk factors. The increase in prescription rate of certain types of medication is not per se due to the introduction of the LTA, but also because of new insights into drug use. In both cohorts almost all medication is prescribed more frequently after stroke than before stroke. However, only for cholesterol-lowering medication and antithrombotic medication is this increase significant. The increase in use of statins is probably because they are known to be effective in stroke patients, regardless of cholesterol levels [27]. Furthermore, significantly more ACE inhibitors are prescribed after stroke in cohort 2; this might be because ACE inhibitors are known to very effective in the treatment of hypertension [28].

Overall, this study showed only minimal differences between cohort 1 and 2. A possible reason for this is that there were no major differences in the groups before introduction of the LTA. Another explanation could be that there was already a shift in the referral and treatment patterns in stroke.

This study has several limitations. The study population was selected from general practices in the northern part of the Netherlands. To be more representative, the study group should be selected from multiple general practices throughout the Netherlands.

Furthermore, this registry study and its design were not suitable to examine a causal relationship between the introduction of LTA and observed changes in primary and secondary outcomes.

In 2003 in the Netherlands a new healthcare system was introduced and (amongst other changes) required more accurate registration of data. Cohort 1 was selected before these changes and cohort 2 after these changes were introduced. Therefore, some results may be due to improved registration rather than to the influence of the LTA. However, in the present study this is accounted for by, for example, not examining the number of prescriptions written by the GP because this was hardly registered in cohort 1.

It should also be noted that registration in the RNG depends on the personal preference of the GP. The ICPC codes do not measure in absolute terms what they intend to measure. A follow-up study should not only use the dataset, but should also send questionnaires to the patients so that all details from the medical records can be verified.

## Conclusion

The ‘Dutch Transmural Protocol TIA/CVA’ was introduced to improve survival after stroke and minimize the risk of stroke recurrence. No major change in one-year survival was found after introduction of the LTA. For the secondary outcomes, a significant increase was found in the number of patients diagnosed with of hypercholesterolemia and in prescriptions of ACE inhibitors. Also, a slight increase was observed in the number of contacts with the nurse practitioner. However, these results may be due to the introduction of the LTA and/or to other causes.

### Practice implications

This study shows that, even after introduction of the LTA, the ideal treatment has not yet been achieved. Improvements might be made by more stringent follow-up of the LTA in general practice. Future research should focus on, e.g., using questionnaires to obtain more data on implementation of the LTA by GPs, and to what extent it is found to be effective. Also, a longer follow-up period might reveal a significant difference in survival.

## Competing interests

All authors declare that they have no competing interests.

## Authors’ contributions

LW, FG, BK and KM initiated the study. LW wrote the protocol. FG and KM supervised data collection. LW, FG, BK and KM wrote the manuscript. LW and BK performed statistical analysis. All the authors have read and reviewed the final manuscript.

## Pre-publication history

The pre-publication history for this paper can be accessed here:

http://www.biomedcentral.com/1471-2296/14/74/prepub
